# Hospital competition and the expenses for treatments of acute and non-acute common diseases: evidence from China

**DOI:** 10.1186/s12913-019-4543-x

**Published:** 2019-10-22

**Authors:** Chenhui Deng, Jay Pan

**Affiliations:** West China School of Public Health and West China Fourth Hospital, Sichuan University, No. 17, Section 3, Ren Min Nan Road, Chengdu, 610041 Sichuan China

**Keywords:** Hospital competition, Market concentration, Medical expense, Health reform

## Abstract

**Background:**

Because there is heterogeneity in disease types, competition among hospitals could be influenced in various ways by service provision for diseases with different characteristics. Limited studies have focused on this matter. This study aims to evaluate and compare the relationships between hospital competition and the expenses of prostatectomies (elective surgery, representing treatments of non-acute common diseases) and appendectomies (emergency surgery, representing treatments of acute common diseases).

**Methods:**

Multivariable log-linear models were constructed to determine the association between hospital competition and the expenses of prostatectomies and appendectomies. The fixed-radius Herfindahl-Hirschman Index was employed to measure hospital competition.

**Results:**

We collected data on 13,958 inpatients from the hospital discharge data of Sichuan Province in China from September to December 2016. The data included 3578 prostatectomy patients and 10,380 appendectomy patients. The results showed that greater competition was associated with a lower total hospital charge for prostatectomy (*p* = 0.006) but a higher charge for appendectomy (*p* <  0.001). The subcategory analysis showed that greater competition was consistently associated with lower out-of-pocket (OOP) and higher reimbursement for both surgeries.

**Conclusions:**

Greater competition was significantly associated with lower total hospital charges for prostatectomies, while the opposite was true for appendectomies. Furthermore, greater competition was consistently associated with lower OOP but higher reimbursement for both surgeries. This study provides new evidence concerning the heterogeneous roles of competition in service provision for non-acute and acute common diseases. The findings of this study indicate that the pro-competition policy is a viable option for the Chinese government to relieve patients’ financial burden (OOP). Our findings also provide references and insights for other countries facing similar challenges.

## Background

General economic theory suggests that competition drives efficiency (both technical and allocative), which would lower costs [1, 2]. Many countries have introduced pro-competition policies in health care services, aiming to maximize the return of investment [3–5]. These mechanisms range from market-driven policies to a so called “internal market” mechanisms in a strictly-regulated and public-owned system [6]. However, the results are far from conclusive. Claims have been made to two extremes: some studies argue that competition in the health care sector is effective, resulting in lowered medical expenses [7–9], while others failed to reveal an association between hospital competition and medical expenses [10–14].

Variations in market properties and system contexts are often blamed for the inconsistent findings [15]. Unlike the manufacturing and service industries, there often exists a serious lack of consumers’ freedom of choice in many health care systems. Even when choices are made available, consumers still have difficulty in making a rational decision due to the asymmetrical distribution of medical information [16–20]. Most empirical studies investigating the role of competition in health care services have been conducted in the United States (US) and the United Kingdom (UK) [6]. The US health system favors consumer choice and is funded by a variety of health funds (including commercial funds and the government-funded Medicare and Medicaid programs). Empirical evidence has shown that competition reduces costs in the US [6, 21–24]. Compared with the US, the UK has established a universal health care system that features public ownership; however, hospital competition is still encouraged. Evidence concerning the impact of competition on health expenses in the UK is mixed [25, 26].

In line with the international trend, health reforms in China during the past three decades have also witnessed a strong emphasis on the role of competition. However, debates about the outcomes still continue. Some researchers have argued that hospital competition is associated with lower expenses [16, 27–29], while other studies reported insignificant impacts [30].

The hospital system in China is characterized with consumer-driven competition. Thanks to the universal coverage of the government-subsidized social health insurance programs [31, 32], both public and private hospitals are subject to governmental pricing regulations, unless they opt out of the insurance coverage. However, there would be potential “price” competition of total hospital charges in the hospital market in China. The price for each health service item is regulated in China, but the total charge of the treatments for single disease is not fixed. Under the fee-for-service (FFS) payment system in China, the total charge depends on the number of services and their prices. Thus, for the same disease’s treatment, the payments could vary, depending on the services intensity. Seeking a larger market share, a hospital may lower the total hospital charge of the treatments for a certain disease to attract patients who are “price” sensitive. For patients, the total hospital charge consists of their out-of-pocket expenses (OOP) and the reimbursement. OOP includes both health insurance covered services’ copayments and payments for services not covered by health insurance. Although universal health insurance coverage has been achieved in China [31, 32], the services covered by the social health insurance programs are limited [33]. Health services can be divided into two groups: services covered by the social health insurance programs and those that are not. The covered services are co-paid by the patients and the health insurance programs, while those not covered are paid in full by the patients. Thus, patients’ OOP payments not only include the covered services’ copayments, but also the uncovered services’ payments. Moreover, higher pricing and higher OOP payments apply to services delivered by higher levels of hospitals. Consumers enjoy the freedom to choose more expensive services if they are willing to pay a higher OOP fee. This includes bypassing primary care for hospital services. Under such circumstances, hospitals in China are incentivized to attract as many patients as possible to obtain potential financial benefits.

In the face of competition, physicians also have great incentive to deliver excessive health care services under the China’s health care system [33, 34]. A physician’s salary in hospitals in China is a floating wage, mostly depending on the surplus income of the delivered service. Their income consists of two parts: basic salary and performance salary [35]. The basic salary is mainly determined by the physician’s professional title (resident physician, associate chief physician, or chief physician) and working age (length of service). The performance salary is decided based on the financial performance of their clinical department [36, 37]. The physician’s total performance salary will be calculated by the hospital’s financial department monthly according to the department’s surplus (revenue minus cost). The department head is responsible for distributing the performance salaries to the physicians and nurses within the department. In most cases, physicians’ performance income account for more than 80% of their total income [36]. In China, a hospital’s revenue from the treatments of a specific patient is completely equal to the payments from the patient. The payments are co-paid by the patient and their health insurance program. Under the FFS payment system, the hospital’s revenue from each patient varies according to the number of services it is providing and the prices of the services, thus providing physicians with great incentives to provide excessive health care services.

Due to a lack of autonomy and the large amount of asymmetric information between health care providers and patients [16–20], competition in the hospital market may not lower the “price” (total hospital charges), compared with the general market. It may even lead to higher total hospital charges due to more supplier-induced demand. However, a lack of autonomy and abundant asymmetric information between health care providers and patients might not be the case with the treatments of all the diseases. If the unique properties addressed vary among treatments for specific diseases, the competition effect on the total hospital charge would differ.

In this study, we hypothesize that competition would be associated with lower total hospital charges for treatments of non-acute common diseases, but higher charges for treatments of acute common diseases in the context of China. The hypothesis will be further explained in the “Study hypothesis” section.

To test this hypothesis, we chose two common surgically-treated diseases: benign prostatic hyperplasia (BPH) treated with a prostatectomy and acute appendicitis, treated with an appendectomy. Since determining whether the patient is in an acute condition is difficult, we chose surgically-treated diseases to be sure of the onset of the condition. Emergency surgery is the preferred treatment for an acute surgical disease, while elective surgery is preferred for patients with non-acute diseases. The surgical treatments for the two common diseases have less asymmetric information between the health care provider and the patients, but there is a difference in the degree of emergency between the two diseases. Acute appendicitis represents an acute common disease, while BPH represents a non-acute common disease.

**Fig. 1:**
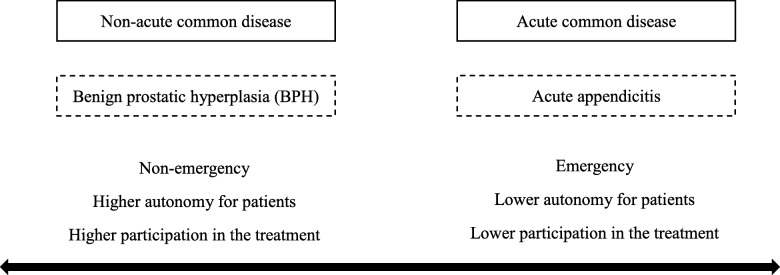
Differences in degree of emergency between acute and non-acute common diseases

Figure 1 represents the differences in the degree of emergency between acute and non-acute common diseases. The acute common disease is on the right, represented by acute appendicitis, while the non-acute disease on the left is represented by benign prostatic hyperplasia. Compared with non-acute common diseases, the acute ones are always more pressing, and patients with acute diseases or the people around them have a lower degree of participation in the clinical therapy plan and treatment, showing low autonomy for the services.

There is a conspicuous gap in the literature concerning the role of competition and its effect on different disease conditions and medical procedures [11, 16, 30, 38]. This study aimed to fill this gap by assessing the associations between competition (market concentration) and medical expenses for elective and emergency surgical procedures.

## Methods

### Study hypothesis

This study hypothesizes that competition would associate with lower total hospital charges for treatments of non-acute common diseases but higher charges for treatments of acute common diseases in the context of China.

Considering that there would be less unique properties in the provision of treatment for non-acute common diseases, competition would play a similarly positive role in the regular services market to effectively mobilize production. For example, enhancing internal management to improve efficiency and to control operation costs and engaging less in the supplier induced demand. As predicted by general economic theory, these competitive strategies would lower the total hospital charges for non-acute common diseases’ treatments. Thus, we assume that competition would be significantly associated with lower total hospital charges for treatments of non-acute common diseases (prostatectomy).

Conversely, patients with acute common diseases have lower autonomy concerning service choice. Their acute symptoms mean that doctors need to operate immediately and does not allow them or their relatives enough time to choose the hospital, unlike the opportunities available for non-acute common diseases. Faced with a higher degree of competition, to achieve their financial goals, care providers of patients with acute common diseases may utilize the patient’s disadvantages in lack of autonomy. Hospitals would then engage in more supplier-induced demand, rather than adopting the competitive behavior of improving efficiency and controlling operation costs. It can therefore be assumed that competition would be significantly associated with higher total hospital charges for treatments of acute common diseases (appendectomy).

### Setting

This study was conducted in Sichuan province in the southwest of China. Sichuan ranks fifth in land size (486,052 km^2^) and forth in population size (83 million) in China [39]. Moreover, three large disparities in socioeconomic development exist in Sichuan. The eastern part of Sichuan is composed of mostly plains and enjoys flourishing economic growth and high population density. In contrast, the western part of Sichuan is characterized by mountainous areas, poor economy, and a sparse population [40]. These regional differences have resulted in a high level of variation in available medical resources, particularly concerning the market concentration of hospitals, providing opportunities to identify the association between hospital competition and medical expenses.

### Data

Data were extracted from the Sichuan Provincial Health Statistics Support System Database (SPHSSSD), covering the period of 4 months, from September 1, 2016, to December 31, 2016. All hospitals are required to submit their data—a minimal dataset about their admitted patients and patient care services—to the SPHSSSD. The data include the demographic characteristics of patients, principal and additional diagnoses, procedures performed, and medical expenses charged to both the insurance funds and to the patient.

Patient data collected by the SPHSSSD were linked to a unique hospital identification code. We collected further information about each of the hospitals from the hospitals’ annual reports. The latitude and longitude of each hospital was identified in the geographic information system for the purpose of estimating market concentration.

We restricted the study subjects to those who underwent surgical procedures for BPH and acute appendicitis to represent patients with non-acute and acute common diseases for several reasons.^1^ First, these two procedures are commonly performed in hospitals, with the former always being elective and the latter being urgent or emergency treatment. It was estimated that in 2015, 105 million men globally suffered from BPH, compared with 11.6 million patients that suffered from acute appendicitis [41]. After the age of 80 years, approximately 90% of men are affected by BPH [42]. Second, the two conditions involved different degrees of consumer participation and choice in clinical decision making. Acute appendicitis represents an urgent condition, meaning that the doctor must make a prompt decision about whether an operation is needed. Contrastingly, BPH represents a non-urgent condition. The decision of surgical procedure is usually made by the patient while considering a range of socioeconomic factors and clinical options. Third, death rates associated with the surgical procedures for the two conditions are very low. Approximately 50,100 out of 11.6 million patients undergoing appendectomies died [43]. It is even more rare for patients to die during a simple prostatectomy.

**Fig. 2:**
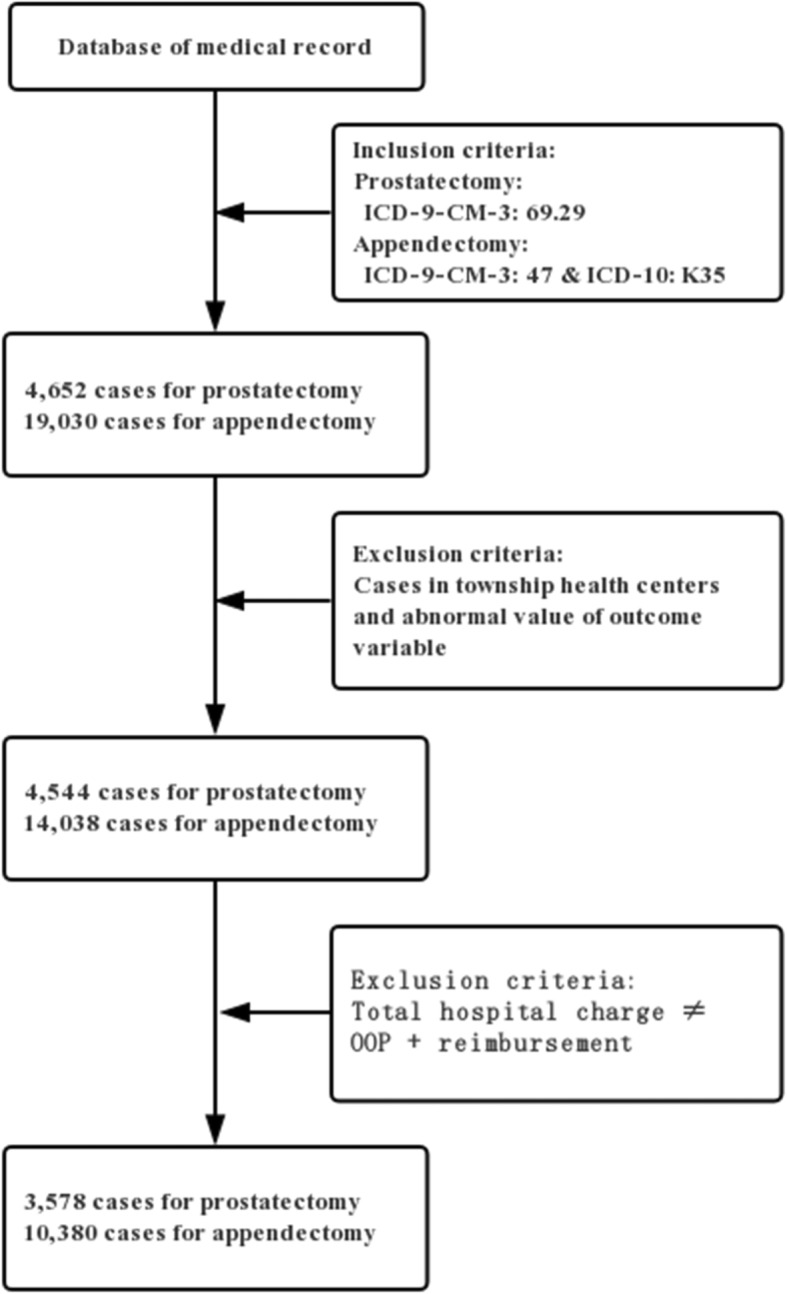
Flowchart of the determination of study population

Figure 2 shows a flowchart of the sample selection in detail. The samples were identified using the International Classification of Diseases, 9th Revision, Clinical Modification (ICD-9-CM) and/or the International Classification of Diseases, 10th Revision (ICD-10) procedure codes. Prostatectomy was identified by ICD-9-CM code 60.29, whereas appendectomy was identified by the ICD-9-CM code of 47 and ICD-10 code of K35. A total of 13,958 inpatients—including 3578 who underwent prostatectomies and 10,380 who underwent appendectomies—from 495 hospitals in Sichuan Province within the study period were included in the final empirical analysis.

### Variables

Three main outcome variables were selected: total hospital charge and its two components: OOP expenses and reimbursement. Because the three expense indicators’ distributions were highly right-skewed, they were log transformed in the regression analysis.

The key explanatory variable was the Herfindahl-Hirschman Index (HHI) [44, 45], which measures the degree of hospital competition (market concentration). The HHI is widely used in related studies [46, 47]. The smaller the HHI, the more competitive (or less concentrated) the market is. Referring to previous literature, the fixed-radius method was employed to define the hospital market by a 15-mile radius around each hospital [44, 48]. Next, the HHI was calculated by adding together the squares of the fraction of the number of specified surgery procedures from each hospital in the market.

We also included the following control variables in the regression analysis: patient gender, age, occupation, insurance program, admission severity, whether in critical condition, admission approach, length of stay, hospital classification, hospital level, profit and ownership classification, urbanization rate, and the GDP per capita in the hospital’s county. Lastly, we included a set of county dummies to consider the regional invariant factors.

### Statistical analysis

The continuous variables were examined as medians with interquartile range (IQR), whereas the categorical variables were described using frequency in the descriptive analysis.

A multivariable log-linear model was used to analyze the association of hospital competition with the two common surgery expenses. The model was set as follows:

1 $$ \log {(Expense)}_{ihc}=\alpha {HHI}_{hc}+{\mathbf{Individual}}_{\mathbf{ihc}}\boldsymbol{\upbeta} +{\mathbf{Hospital}}_{\mathbf{hc}}\boldsymbol{\uplambda} +{\mathrm{Region}}_{\mathrm{c}}\upgamma +{\varepsilon}_{ihc} $$

where *i* donates the inpatient, *h* donates the hospital, and *c* donates the county. Log (*Expense*) is the outcome variable and represents the log transformation of inpatient expense. *HHI* is the key explanatory variable, measuring the degree of hospital competition. ***Individual*** represents the explanatory variables of the inpatient characteristics, including gender, age, occupation, insurance programs, admission severity, whether in critical condition, admission approach (emergency service, outpatient service, or referral arrangement), and length of stay. ***Hospital*** refers to the explanatory variables of the hospital’s characteristics, including hospital classification, hospital level, and profit and ownership classification. ***Region*** refers to the urbanization rate, the GDP per capita, and a set of dummy variables of counties. *ε* is the error term.

The key coefficients of interest are represented by *α*, which captures the relationship between hospital competition and inpatient expenses. A positive estimation of *α* indicates that a higher hospital competition degree (lower value of HHI) is associated with decreasing expenses, whereas the negative estimation shows that hospital competition is associated with increasing expenses. ***β***, ***λ***, and ***γ*** measure the change in the outcome variable in an individual’s, hospital’s, and regional characteristics respectively.

Statistical Analysis System (SAS) software version 9.4 and R software were used for the statistical analysis. Statistical significance was set at *p* <  0.05.

## Results

### Descriptive statistics

Table 1 shows the demographics and clinical characteristics of inpatients and hospitals. The median total hospital charge for inpatients undergoing prostatectomy and appendectomy were ¥ 13,775 (IQR: ¥ 11,118–¥ 17,299) and ¥7469 (IQR: ¥ 5733–¥ 10,076) respectively. The median HHI was 0.38 (IQR: 0.22–0.65) in the prostatectomy market and 0.18 (IQR: 0.07–0.37) in the appendectomy market, showing that the appendectomy market was generally more competitive (or less concentrated) than that of prostatectomy.

**Table 1:** Demographics and clinical characteristics of inpatients and hospitals^a^

	Surgical Group	
Characteristics	Prostatectomy (*n* = 3578)	Appendectomy (*n* = 10,380)
Total hospital charges median (IQR),¥	13,775 (11118–17,299)	7469 (5733–10,076)
Mean ± standard deviation,¥	14,891 ± 6178	8389 ± 4228
Out-of-pocket expense median (IQR),¥	2253 (0–1793)	1949 (0–3290)
Mean ± standard deviation,¥	2253 ± 5044	1949 ± 3380
Reimbursement median (IQR),¥	12,691 (8809–16,469)	6217 (2971–9263)
Mean ± standard deviation,¥	12,639 ± 7741	6440 ± 5304
HHI median (IQR)	0.38 (0.22–0.65)	0.18 (0.07–0.37)
Mean ± standard deviation	0.43 ± 0.28	0.27 ± 0.28
Gender		
Male	3578 (100%)	5395 (51.97%)
Female	0 (0%)	4984 (48.02%)
Unknown	0 (0%)	1 (0.01%)
Age median (IQR), years	71 (65–76)	45 (31–59)
mean ± standard deviation, years	71 ± 9	45 ± 17
Occupation		
Civil servant	23 (0.64%)	73 (0.70%)
Office worker	38 (1.06%)	484 (4.66%)
Worker	33 (0.92%)	371 (3.57%)
Farmer	1124 (31.42%)	3080 (29.67%)
Unemployed	98 (2.74%)	219 (2.12%)
Retired	437 (12.21%)	172 (1.66%)
Unknown	1825 (51.01%)	5981 (57.62%)
Insurance program		
Urban employees’ basic medical insurance	948 (26.50%)	2072 (19.96%)
Urban residents’ basic medical insurance	682 (19.06%)	1845 (17.77%)
New rural co-operative medical insurance	1342 (37.51%)	3897 (37.54%)
Full public expense	34 (0.95%)	164 (1.58%)
Not insured	240 (6.71%)	1229 (11.84%)
Other insurance	332 (9.27%)	1173 (11.31%)
Admission severity		
Emergency	26 (0.72%)	140 (1.35%)
Critical	343 (9.59%)	4498 (43.33%)
General	3209 (89.69%)	5742 (55.32%)
Whether in critical condition		
Yes	260 (7.27%)	70 (0.68%)
No	3308 (92.45%)	9606 (92.54%)
Unknown	10 (0.28%)	704 (6.78%)
Admission approach		
Emergency service	492 (13.75%)	4257 (41.01%)
Outpatient service	2812 (78.59%)	5064 (48.79%)
Referral arrangement	15 (0.42%)	13 (0.12%)
Other	259 (7.24%)	1046 (10.08%)
Length of stay median (IQR), days	13 (11–17)	7 (5–8)
mean ± standard deviation, days	15 ± 6	7 ± 3
Hospital classification		
Special hospitals	117 (3.27%)	157 (1.51%)
General hospitals	3458 (96.65%)	10,217 (98.43%)
Minority hospitals	3 (0.08%)	6 (0.06%)
Hospital level		
Primary	16 (0.45%)	79 (0.76%)
Secondary	857 (23.95%)	4529 (43.63%)
Tertiary	2634 (73.62%)	5566 (53.62%)
Ungraded	71 (1.98%)	206 (1.98)
Profit and ownership classification		
Public (all non-profit)	3407 (95.22%)	9602 (92.51%)
Non-profit private hospital	66 (1.84%)	376 (3.62%)
For-profit private hospital	105 (2.94%)	402 (3.87%)
Urbanization rate median (IQR), %	51.42 (37.17–73.42)	48.97 (37.20–67.63)
mean ± standard deviation, %	57.55 ± 21.92	55.35 ± 22.13
GDP per capita median (IQR),¥	40,609 (28561–61,177)	40,609 (28698–66,235)
mean ± standard deviation,¥	46,349 ± 23,701	47,248 ± 24,343

Abbreviations: *HHI* Herfindahl-Hirschman Index; *IQR* interquartile range

^a^Unless otherwise indicated, data are expressed as weighted numbers (percentages) of row totals of each surgical group. Estimated counts were rounded to the nearest unit

The median ages of the inpatients undergoing prostatectomies and appendectomies were 71.00 years (IQR: 65.00–76.00 years) and 45.00 years (IQR: 31.00–59.00 years) respectively. The inpatients undergoing a prostatectomy were all male (100%), whereas the gender proportion was nearly 1:1 (51.97% [*n* = 5395] male and 48.02% [*n* = 4984] female) for inpatients undergoing an appendectomy. Except for the “Unknown” group, “farmer” was the most common occupation for inpatients (31.42% [*n* = 1124] for prostatectomy and 29.67% [*n* = 3080] for appendectomy). The New Rural Cooperative Medical Care was the most common insurance type in our sample. Of the sample, 37.51% (*n* = 1342) and 37.54% (*n* = 3897) of inpatients undergoing prostatectomy and appendectomy respectively were subscribed to this insurance. The median length of stay of inpatients undergoing prostatectomy (13 days) was longer than that of inpatients undergoing appendectomy (7 days).

The most popular hospitals were general (96.65% [*n* = 3458] for prostatectomy and 98.43% [*n* = 10,217] for appendectomy), tertiary-level (73.62% [*n* = 2634] for prostatectomy and 53.62% [*n* = 5566] for appendectomy), and public hospitals (95.22% [*n* = 3407] for prostatectomy and 92.51% [*n* = 9602] for appendectomy).

### Regression results

Table 2 illustrates the relationship between hospital competition and total hospital charge after adjusting for all of the covariables. The results showed the inverse associations of hospital competition with prostatectomy and appendectomy expenses. Competition was significantly and negatively associated with total hospital charge for inpatients undergoing prostatectomy. Every 10% increase in competition (0.1 unit decrease of HHI value) was associated with an average reduction of 2.10% in the total hospital charge (*p* = 0.006). However, competition was significantly and positively associated with the total hospital charge for appendectomy, with every 10% increase in competition associated with an average increase in hospital charge of 1.27% (*p* <  0.001).

**Table 2:** Results of multivariable log-linear models of total hospital charge for prostatectomy and appendectomy patients

	Surgical Group			
	Prostatectomy		Appendectomy	
Factor	Proportional Change (95% CI)	*P* Value	Proportional Change (95% CI)	*P* Value
HHI	0.210	0.006	−0.127	< 0.001
	(0.059, 0.361)		(− 0.196, − 0.058)	
Gender				
Male	NA	NA	1[Reference]	NA
Female	NA	NA	−0.005	0.36
			(−0.014, 0.005)	
Age	0.003	< 0.001	0.003	< 0.001
	(0.002, 0.003)		(0.002, 0.003)	
Occupation				
Unemployed	1[Reference]	NA	1[Reference]	NA
Civil servant	−0.083	0.11	0.029	0.42
	(−0.184, 0.018)		(−0.041, 0.098)	
Office worker	0.060	0.21	−0.008	0.72
	(−0.034, 0.154)		(−0.054, 0.037)	
Worker	0.035	0.46	−0.014	0.55
	(−0.058, 0.127)		(−0.061, 0.032)	
Farmer	−0.013	0.65	−0.019	0.34
	(−0.071, 0.044)		(−0.059, 0.020)	
Retired	0.001	0.97	0.019	0.49
	(−0.058, 0.061)		(−0.035, 0.073)	
Unknown	−0.022	0.44	0.006	0.76
	(−0.079, 0.034)		(−0.032, 0.044)	
Insurance program				
Not insured	1[Reference]	NA	1[Reference]	NA
Urban residents’ basic medical insurance	−0.002	0.92	−0.023	0.02
	(−0.034, 0.031)		(−0.043, − 0.003)	
Urban employee basic medical insurance	0.002	0.91	−0.034	<.001
	(−0.029, 0.033)		(−0.053, − 0.015)	
Full public expense	−0.086	0.03	−0.027	0.22
	(−0.165, 0.008)		(−0.070, 0.016)	
New rural co-operative medical insurance	−0.015	0.33	−0.017	0.08
	(−0.047, 0.016)		(−0.036, 0.002)	
Others	−0.018	0.33	−0.020	0.07
	(−0.057, 0.019)		(−0.042, 0.002)	
Admission severity				
Emergency	1[Reference]	NA	1[Reference]	NA
Critical	−0.009	0.86	−0.129	< 0.001
	(−0.112, 0.094)		(−0.174, − 0.085)	
General	0.006	0.90	−0.163^***^	< 0.001
	(−0.095, 0.108)		(−0.208, − 0.118)	
Critical condition				
No	1[Reference]	NA	1[Reference]	NA
Yes	0.087	< 0.001	0.101	< 0.001
	(0.054, 0.120)		(0.079, 0.123)	
Admission approach				
Emergency service	1[Reference]	NA	1[Reference]	NA
Outpatient service	−0.011	0.39	−0.013	0.04
	(− 0.036, 0.014)		(− 0.025, − 0.001)	
Referral arrangement	0.177	0.001	0.065	0.35
	(0.068, 0.286)		(−0.072, 0.202)	
Other	−0.010	0.62	0.011	0.39
	(−0.050, 0.030)		(−0.014, 0.036)	
Length of stay	0.029	< 0.001	0.055	< 0.001
	(0.028, 0.030)		(0.053, 0.056)	
Hospital classification				
Special hospitals	1[Reference]	NA	1[Reference]	NA
General hospitals	0.045	0.19	0.063	0.03
	(−0.022, 0.112)		(0.006, 0.121)	
Minority hospital	0.118	0.39	0.121	0.34
	(−0.152, 0.388)		(−0.127, 0.368)	
Hospital level				
Primary	1[Reference]	NA	1[Reference]	NA
Secondary	0.074	0.32	0.290	< 0.001
	(−0.072, 0.221)		(0.225, 0.356)	
Tertiary	0.207	0.008	0.559	< 0.001
	(0.054, 0.360)		(0.491, 0.627)	
Ungraded	−0.065	0.39	0.125	< 0.001
	(−0.214, 0.084)		(0.052, 0.197)	
Profit and ownership classification				
Public (all non-profit)	1[Reference]	NA	1[Reference]	NA
Non-profit private hospital	−0.230	< 0.001	0.189	< 0.001
	(−0.299, − 0.161)		(− 0.220, − 0.157)	
For-profit private hospital	− 0.237	< 0.001	0.182	< 0.001
	(−0.308, − 0.166)		(− 0.217, − 0.147)	
Urbanization rate	− 0.876	< 0.001	− 0.051	< 0.001
	(− 1.202, − 0.559)		(− 0.077, − 0.024)	
GDP per capita	0.001	< 0.001	0.00006	< 0.001
	(0.0007, 0.0013)		(0.00003, 0.00009)	
County dummies	Yes		Yes	

Abbreviations: *NA* not applicable; *HHI* Herfindahl-Hirschman Index; *CI* confidence interval

The results in Table 2 show that the inpatients’ age and the length of their stay were positively associated with the total hospital charge for both surgery types. There was no statistically significant difference in the total hospital charge between inpatients of different occupations. Compared with public hospitals, the total hospital charges of private hospitals (both non-profit and for-profit private hospitals) were lower for inpatients undergoing prostatectomies but higher for inpatients undergoing appendectomies.

In China, the goal of basic health insurance with universal coverage was achieved in 2010. Since then, basic health insurance programs have been playing greater roles in health care financing. The total hospital charge is composed of OOP expenses and insurance reimbursement. We therefore further analyzed the associations between hospital competition and OOP and reimbursement expenses. Table 3 reports the regression estimations of the relationships between hospital competition and OOP and reimbursement after adjusting for all of the covariables.

**Table 3:** Regression results considering hospital competition and OOP and reimbursement after adjusting for all covariables

Factor	Surgical Group	OOP		Reimbursement	
		Proportional Change (95% CI)	*P* Value	Proportional Change (95% CI)	*P* Value
HHI	Prostatectomy	7.141 (5.393, 8.889)	< 0.001	−3.505 (−5.128, − 1.882)	< 0.001
	Appendectomy	0.920 (0.245, 1.596)	0.008	−1.283 (− 1.959, − 0.608)	< 0.001
Control Variables^a^		Yes		Yes	

Abbreviations: *OOP* out of pocket; *HHI* Herfindahl-Hirschman Index; *CI* confidence interval

^a^Adjusted by the following control variables: gender, age, occupation, insurance, admission severity, critical condition, admission approach, length of stay, hospital classification, hospital level, profit and ownership classification, urbanization rate, GDP per capita, and a set of county dummies

Contrary to the different relationships between the two surgeries of competition and total expenses, the estimations of the sub-expenses showed that there were consistent associations with OOP and reimbursements. Hospital competition was significantly and negatively associated with OOP expenses, whereas it was significantly and positively associated with reimbursement for both common surgically-treated diseases. For inpatients undergoing a prostatectomy, every 10% increase in competition (0.1 unit decrease of HHI value) was associated with an average reduction of 71.41% (*p* <  0.001) in OOP expenses and a 35.05% increase in reimbursement (*p* <  0.001). For inpatients undergoing an appendectomy, it was associated with an average 9.2% reduction in OOP expenses of 9.2% (*p* = 0.008) and a 1.28% increase in reimbursement (*p* <  0.001).

Hospitals in China are divided into three levels: primary hospitals, secondary hospitals, and tertiary hospitals. The higher the hospital level is, the larger the scale and the better quality of care is offered [49]. Considering that different level hospitals would respond differently to competition, we further added the interaction term of HHI and hospital level in the regression to explore this. Table 4 presents the estimation results. The results showed that the total hospital charges for inpatients undergoing a prostatectomy in secondary and tertiary hospitals were, on average, 47.3 and 69.3% higher than in the primary hospitals respectively. For patients who underwent appendectomies, the total hospital charges in secondary and tertiary hospitals were, on average, 26.0 and 59.4% higher than in primary hospitals respectively. The interesting finding concerning the interaction terms is that the competition effects on the total hospital charges for patients who underwent prostatectomies are attenuated with higher levels of hospitals: 10% higher competition (10% lower in HHI) correlates with a 9.92, 2.98, and 0.2% reduction in total hospital charges for patients who underwent prostatectomy in primary, secondary, and tertiary hospitals respectively. However, for appendectomies, the competition effects do not seem to differ between hospitals levels.

**Table 4:** Results of multivariable log-linear models of total hospital charge considering hospital level and interaction term of HHI and hospital level

Factor	Appendectomy		Prostatectomy	
	Proportional Change (95% CI)	*P* Value	Proportional Change (95% CI)	*P* Value
HHI	−0.344	< 0.001	0.992	< 0.001
	(−0.517, − 0.237)		(0.407, 1.576)	
Hospital level				
Primary	1[Reference]		1[Reference]	
Secondary	0.260	< 0.001	0.473	0.006
	(0.166, 0.355)		(0.134, 0.812)	
Tertiary	0.594	< 0.001	0.693	< 0.001
	(0.497, 0.691)		(0.354, 1.032)	
Ungraded	0.089	0.099	0.197	0.262
	(−0.017, 0.196)		(−0.147, 0.542)	
HHI*Hospital level				
HHI*Primary hospital	1[Reference]		1[Reference]	
HHI*Secondary hospital	0.282	0.136	−0.694	0.032
	(−0.089, 0.652)		(−1.329, − 0.060)	
HHI*Tertiary hospital	−0.051	0.783	−0.990	0.002
	(− 0.415, 0.313)		(−1.608, − 0.373)	
HHI*Ungraded hospital	0.287	0.161	−0.309	0.352
	(−0.114, 0.688)		(−0.961, 0.343)	
Control variables^a^	Yes		Yes	

Abbreviations: *HHI* Herfindahl-Hirschman Index; *CI* confidence interval

^a^Adjusted by the following control variables: gender, age, occupation, insurance, admission severity, critical condition, admission approach, length of stay, hospital classification, hospital level, profit and ownership classification, urbanization rate GDP per capita and a set of county dummies

### Sensitivity analysis

To test the robustness of our results, we conducted two more sensitivity analyses.

First, HHI was used as a continuous variable in our main analysis—assuming the linearity of the relationship between HHI and expense—to fully utilize the variation information in HHI. However, considering a potentially non-linear relationship, an alternative method is to transform HHI into a categorical variable [10]. To examine the sensitivity, we therefore used the HHI as a categorical variable in the regressions with the same setting with our main analysis. According to the literature, an HHI greater than or equal to 0.25 was categorized as noncompetitive (highly concentrated), between 0.15 and 0.25 as moderately competitive (moderately concentrated), and less than 0.15 as highly competitive (unconcentrated) [10]. The new estimations—the results of which are reported in Table 5—show a similar trend to our main analysis.

**Table 5:** Association between hospital competition and total hospital charge with HHI as a categorical variable

	Surgical Group			
	Prostatectomy		Appendectomy	
Factors	Proportional Change (95% CI)	*P* Value	Proportional Change (95% CI)	*P* Value
Hospital Competition				
Insufficient (high concentrated, HHI ≧ 0.25)	1[Reference]	NA	1[Reference]	NA
Moderate (moderately concentrated, 0.15 < HHI < 0.25)	−0.016	0.62	0.051	0.009
	(−0.048, 0.081)		(0.013, 0.089)	
High (unconcentrated, HHI ≦0.15)	−0.642	< 0.001	0.090	< 0.001
	(−1.019, −0.265)		(0.046, 0.134)	
Control Variables^a^	Yes		Yes	

Abbreviations: *NA* not applicable, *HHI* Herfindahl-Hirschman Index; *CI* confidence interval

^a^Control variables: gender, age, occupation, insurance, admission severity, whether in critical condition, admission approach, length of stay, hospital classification, hospital level, profit and ownership classification, urbanization rate, GDP per capita and a set of county dummies

Second, we used the hospital number as an alternative market concentration measure [44]. The results are presented in Table 6. The estimations are similar to that of our main analysis, confirming the robustness of the results.

**Table 6:** Hospital competition and prostatectomy/appendectomy medical expenses using hospital number to measure hospital competition

Factor	Surgical Group	Total hospital charge		OOP		Reimbursement	
		Proportional Change (95% CI)	*P* Value	Proportional Change (95% CI)	*P* Value	Proportional Change (95% CI)	*P* Value
Hospital number	Prostatectomy	−0.003	< 0.001	−0.144	0.004	0.090	0.03
		(−0.004, − 0.001)		(− 0.242, − 0.047)		(0.007, 0.165)	
	Appendectomy	0.004	< 0.001	−0.029	< 0.001	0.039	< 0.001
		(0.003, 0.006)		(−0.040, − 0.019)		(0.028, 0.050)	
Other control variables^a^		Yes		Yes		Yes	

Abbreviations: *OOP* out of pocket; *CI* confidence interval

^a^Adjusted by following control variables: gender, age, occupation, insurance, admission severity, whether in critical condition, admission approach, length of stay, hospital classification, hospital level, profit and ownership classification, urbanization rate, GDP per capita and a set of county dummies

## Discussion

This study evaluated and compared the associations between hospital competition (market concentration) and the expenses for treatments of acute and non-acute common diseases, which were respectively represented by BPH treated with a prostatectomy (elective surgery) and acute appendicitis treated with an appendectomy (emergency surgery) in China.

The results showed that a more competitive hospital market was consistently associated with lower out-of-pocket (OOP) expenses but higher reimbursements for both surgeries, showing that hospitals had a similar competitive strategy in delivering both of the surgery services: lowering the OOP to attract patients while gaining more from the health insurance programs to achieve their financial goals.

The total hospital charge is composed of OOP expenses and reimbursement. With the universal health insurance coverage in China [31, 32], patients are more concerned about OOP expenses than the total hospital charge [50]. Therefore, lowering the OOP would be an effective means of attracting patients.

An interesting finding is that hospitals seem to shift the expenses from the patient to the health insurance programs to reduce the OOP while charging more from the health insurance programs. It would be practical and reasonable for the hospitals to achieve the charge shift within China’s current health system context. China achieved their goal of universal health insurance coverage in 2010, but the benefit package provided by social health insurance programs is not generous. Limited drugs and health care services are covered by the social health insurance programs. Competition would provide an incentive for hospitals to use more drugs and services covered by the health insurance programs than those not covered. This would lead to a decrease in OOP expenses and an increase in reimbursements.

However, excepting the reasonable shift, there is a possibility that hospitals may over-deliver drugs and services covered by the health insurance programs. Faced with increasing competition, hospitals would use their information advantage and dominant role in the clinical therapy plan and treatment to deliver unnecessary but reimbursable drugs or services to benefit more from insurance funds. In this case, the increment in reimbursements would even exceed rather than just compensate for the loss in OOP. Following our hypothesis, the expense shifting would differ in the delivery of services concerning acute and non-acute common diseases due to the different degrees in asymmetric information.

The results concerning total hospital charges confirm our hypothesis. The total charges for treatments of an acute common disease—represented by acute appendicitis treated with appendectomy—is positively associated with competition. It is, however, negatively associated with non-acute common diseases, represented by BPH treated with prostatectomy. Furthermore, the absolute estimations of the HHI coefficients in total hospital charge, OOP, and reimbursement are all relatively larger for prostatectomy than for appendectomy, showing that hospitals are more sensitive to competition in delivering services for non-acute common diseases than for acute ones.

### Policy implications

Solving the problem of “*Kan Bing Gui* (expensive medical expenses)” is one of the main goals of China’s new round of national health reform. Consistent with previous research [51], the findings of this study indicate that a pro-competition policy is a viable option by which the Chinese government could relieve patients’ financial burden (OOP).

However, governments should be cautious when introducing the pro-competition policy in the hospital market. Greater competition would lead to lower total hospital charge for non-acute diseases’ services, but higher for acute diseases. Considering society as a whole, competition would potentially exacerbate the health care cost inflation for acute diseases. Finding a feasible approach to monitor hospitals’ behavior would be the primary challenge in implementing the pro-competition policy in the health care market, especially concerning acute diseases. Our findings provide references and insights not only for China, but also for countries with a similar health system context.

#### Contributions and limitations

This study contributes to the existing literature in being among the first few studies to explore the heterogeneous roles of competition in health care delivery.

This study has several limitations. First, the health care quality in the analysis was not well controlled due to data limitations. Health care quality is related to both expenses (outcome variable) [52] and hospital competition (explanatory variable). This limitation may result in omitted variable bias. Second, the results of this study cannot be generalized to the effect of competition on the charges of severe diseases. Therefore, the role of competition in severe diseases is still in need of further study. Third, the estimation in this study would be interpreted as correlational rather than causal. Panel data or instrumental variable methods should be employed in a future study to identify the causal effect.

## Conclusions

Greater competition (lower hospital market concentration) was significantly associated with lower total hospital charges for prostatectomy (representing treatments of non-acute common diseases) but higher expenses for appendectomy (representing treatments of acute common diseases). Furthermore, greater competition was consistently associated with a lower OOP but higher reimbursement for both surgeries. The results support our hypothesis that competition would play different roles in the services provision for non-acute and acute diseases.

## Data Availability

The data that support the findings of this study are available from the Health Commission of Sichuan Province but restrictions apply to the availability of these data, which were used under license for the current study, and so are not publicly available. Data are however available from the authors upon reasonable request and with permission of the Health Commission of Sichuan Province.

## Abbreviations

HHI: Herfindahl-Hirschman Index
ICD-10: International Classification of Diseases, 10th Revision
ICD-9-CM: International Classification of Diseases, 9th Revision, Clinical Modification
IQR: Interquartile range
NA: Not applicable
OOP: Out-of-pocket
SAS: Statistical Analysis System
SPHSSSD: Sichuan Provincial Health Statistics Support System Database

## Footnotes

1 Except for the two surgeries reported in the main text, we also analyzed two other elective surgeries (ligation of hemorrhoids and excision of pterygium) and two emergency surgeries (reduction of humeral fracture and inguinal hernia repair) for robust tests. The estimations of the four surgeries showed a similar trend to our main analysis. Considering the emergency conditions of the excision of pterygium, the reduction of humeral fracture, and the inguinal hernia repair could not be identified as clearly as prostatectomy and appendectomy, while the inpatient surgery of the ligation of hemorrhoids is mixed with the day surgery in our database. Therefore, we did not include them in the main text. The results are available upon request.
